# Mesenchymal stromal cells contract collagen more efficiently than dermal fibroblasts: Implications for cytotherapy

**DOI:** 10.1371/journal.pone.0218536

**Published:** 2019-07-15

**Authors:** Sarah A. Hilton, Lindel C. Dewberry, Maggie M. Hodges, Junyi Hu, Junwang Xu, Kenneth W. Liechty, Carlos Zgheib

**Affiliations:** 1 Laboratory for Fetal and Regenerative Biology, Department of Surgery, University of Colorado Denver School of Medicine and Children's Hospital Colorado, Aurora, CO, United States of America; 2 Colorado Fetal Care Center, Colorado Institute for Fetal & Maternal Health, Children’s Hospital of Colorado, Aurora, CO, United States of America; National Cancer Institute, UNITED STATES

## Abstract

**Background:**

Stem cell therapy is the next generation a well-established technique. Cell therapy with mesenchymal stem cells (MSC) has been demonstrated to enhance wound healing in diabetic mice, at least partly due to improved growth factor production. However, it is unclear whether MSC can biomechanically affect wound closure. Utilizing the well-established cell-populated collagen gel contraction model we investigated the interactions between MSC and the extracellular matrix.

**Methods:**

Murine fetal liver-derived Mesenchymal Stem Cells (MSCs) or fetal Dermal Fibroblasts (DFs) were cultured in cell–populated collagen gels (CPCGs). The effect of cell density, conditioned media, growth factors (TGF-B1, FGF, PDGF-BB), cytoskeletal disruptors (colchicine, cytochalasin-D), and relative hypoxia on gel contraction were evaluated. Finally, we also measured the expression of integrin receptors and some growth factors by MSCs within the contracting gels.

**Results:**

Our results show that at different densities, MSCs induced a higher gel contraction compared to DFs. Higher cell density resulted in faster and more complete contraction of CPCGs. Cytoskeletal inhibitors either inhibited or prevented MSC-mediated contraction in a dose dependent fashion. Growth factors, conditioned media from both MSC and DF, and hypoxia all influenced CPCG contraction.

**Discussion:**

The results suggest that MSCs are capable of directly contributing to wound closure through matrix contraction, and they are more effective than DF. In addition, this study demonstrates the importance of how other factors such as cell concentration, cytokines, and oxygen tension can provide potential modulation of therapies to correct wound healing impairments.

## Introduction

Diabetes is the leading cause of amputation in the United States. In diabetic patients, chronic wounds represent a serious challenge and could lead to limbs amputation if not treated effectively. The wound healing process is a complex, dynamic, and orchestrated series of events that leads to wound closure. Alterations in cellular function, growth factor production, vascular insufficiency, chronic inflammation, and bacterial load can result in delayed or impaired wound healing.

Among all available therapies, cell therapy is a promising treatment because of its ability to potentially correct several deficits at the same time, such as chemokine and cytokine production, autologous cell recruitment, and matrix protein synthesis and organization. Improved healing of normal and diabetic wounds has been demonstrated following local administration of fibroblasts [1–4] and bone marrow-derived mesenchymal stromal/stem cell [2, 5–14] transplantation. However, how these cells are able to repair the wound is not yet fully understood.

Dermal fibroblasts (DF) represent another example of cell-based therapy for skin wounds. Others have shown that resident DF contribute to wound contraction by migrating into the wound space, interacting with the extracellular matrix (ECM), and generating biomechanical contractile forces via the interaction of the cytoskeleton with membrane bound integrin receptors [15]. We previously showed that injection of multipotent fetal liver-derived murine mesenchymal stromal cells (MSC) in the skin wound of a diabetic mouse model substantially enhanced wound closure [16]. We also showed that MSC-treated diabetic wounds exhibited improved vascularization and increased mRNA for a number of factors, including transforming growth factor- beta 1 (TGF-beta1), epidermal growth factor (EGF), platelet-derived growth factor (PDGF), and stromal cell-derived factor 1-alpha (SDF-1). Moreover, in our preliminary studies, we demonstrated that MSC possess the ability to contract collagen gels *in vitro*. This suggests that in addition to their paracrine effects, MSC may contribute biomechanically to wound contraction and closure [15].

Taking all this into consideration, we hypothesized that MSC-mediated improvements in wound healing may be through similar mechanisms, and that MSC can directly affect wound contraction and closure. To test our hypothesis, we used the well-known Cell-Populated Collagen Gels (CPCGs) technique to measure the ability of MSC to contract a three-dimensional analogue of the ECM [17–19] and compared it to the DF-induced contraction. We also compared MSC to fetal dermal fibroblast-mediated collagen gel contraction, and we examined the influence on contraction dynamics following specific cytoskeletal disruption using colchicine, cytochalasin-D and the addition of soluble factors normally present in the wound environment. To further characterize the capacity of these cells we measured the mRNA expression of several cytokines, chemokines, and adhesion molecules in MSC-populated collagen gels.

## Methods

### Isolation, culture and characterization of MSC

MSC were isolated from fetal livers of embryonic, day-15 gestation, green fluorescent protein (GFP)-expressing C57BL/6TbN (act-GFP) OsbY01 transgenic mice, as previously described [15]. Briefly, fetal livers were mechanically disrupted into a single cell suspension, and red blood cells were lysed with ammonium chloride. The cell suspension was washed twice with phosphate-buffered saline, and plated at 7.5 x 10^5^ cells/cm^2^ in Mesencult Growth media (MGM): Mesencult Basal Medium for Mouse Mesenchymal Stem Cells, supplemented with Mouse Mesenchymal Stem Cell Stimulatory Supplements (StemCell Technologies, Vancouver, Canada). Media was changed every three days until the cells reached 90% confluence (approximately 2 weeks). Adherent cells were detached with 0.05% trypsin/EDTA, enriched for MSC by CD11b immunodepletion, expanded in MGM, and cryopreserved at passage 2. Previous research confirmed this protocol by characterizing these cells by flow cytometric analysis of cell-surface antigen profile, fibroblast-colony forming unit formation, and multipotent capacity for induced osteogenic and adipogenic lineage differentiation [15]. All experiments were performed with MSC from passages 3 through 6.

### Isolation and culture of DF

DF were isolated from age-matched fetal mice by explant culture [15]. Dermal tissue was harvested, sectioned into 1 cm squares, and placed dermal-side-down onto tissue culture dishes. Tissue pieces were covered with sterile coverslips, to maintain contact with the culture surface, and incubated in Dulbecco’s Modified Eagle’s media (DMEM) supplemented with 10% fetal bovine serum (Cambrex, Charles City, IA). After approximately two weeks, cells that migrated from the dermal surface were detached, expanded, and cryopreserved at passage 2. All experiments were performed with DF from passages 4 through 6. Unless otherwise noted, all cell culture reagents were purchased from Invitrogen (Carlsbad, CA).

### Cell Populated Collagen Gels (CPCGs)

CPCGs were prepared as previously reported [20, 21]. Collagen (2mg/ml) was solubilized in 0.1% acetic acid (rat tail Type I collagen, BD Biosciences, Bedford, MA) and maintained on ice. PH was adjusted to physiologic ionic strength of pH 7.6 with 10X Minimum Essential Media (MEM) and 1N NaOH. A concentrated suspension of cells was gently and thoroughly incorporated into the gel to achieve the final cell densities as described below. The cell suspension was quickly added to 12-well dishes (750 ul per well) and polymerized for 2 hours at 37°C. The solidified gels were then gently released into 6-well dishes containing 3 ml of media, and placed into a 5% CO_2_ incubator at 37°C. The medium was changed every three days.

### Experimental protocol

Three different concentrations of MSC and DF were used: 5 x 10^4^ (low density, LD), 1 x 10^5^ (intermediate density, ID), and 2 x 10^5^ (high density, HD) cells/ml. In addition, two co-cultures of MSC and DF gels were examined: 5 x 10^4^ cells/ml of each (total cell concentration of 1 x 10^5^, corresponding to ID of individual CPCGs), and 1 x 10^5^ cells/ml of each (total cell concentration of 2 x 10^5^ cells/ml, corresponding to HD of individual CPCGs). LD- and HD-MSC gels were also cultured in media containing colchicines, a selective disruptor of microtubules, and/or cytochalasin D, a selective disruptor of microfilaments (Sigma-Aldrich, St. Louis, MO). In addition, LD- and HD-MSC gels were incubated in one of the following: MGM (20% serum control), MGM with only 25% of supplements (low serum control, LS-MGM), a 1:1 mixture of MGM and either MSC- or DF-conditioned media (CM), transforming growth factor beta 1 (TGF-beta1, 15 or 1.5 ng/ml, in LS-MGM), platelet-derived growth factor (PDGF-BB, 50 ng/ml, in LS-MGM), or basic fibroblast growth factor (b-FGF, 50 ng/ml, in LS-MGM). CM was obtained from culture dishes of MSC or DF after three days of incubation post-confluence. Finally, LD- and HD-MSC gels were incubated under relative hypoxic conditions (5% O_2_) to mimic chronic wound conditions. Each experimental condition above was performed in quadruplicate (4 gels per condition per experiment).

### Gel contraction measurement

**Fig 1:**
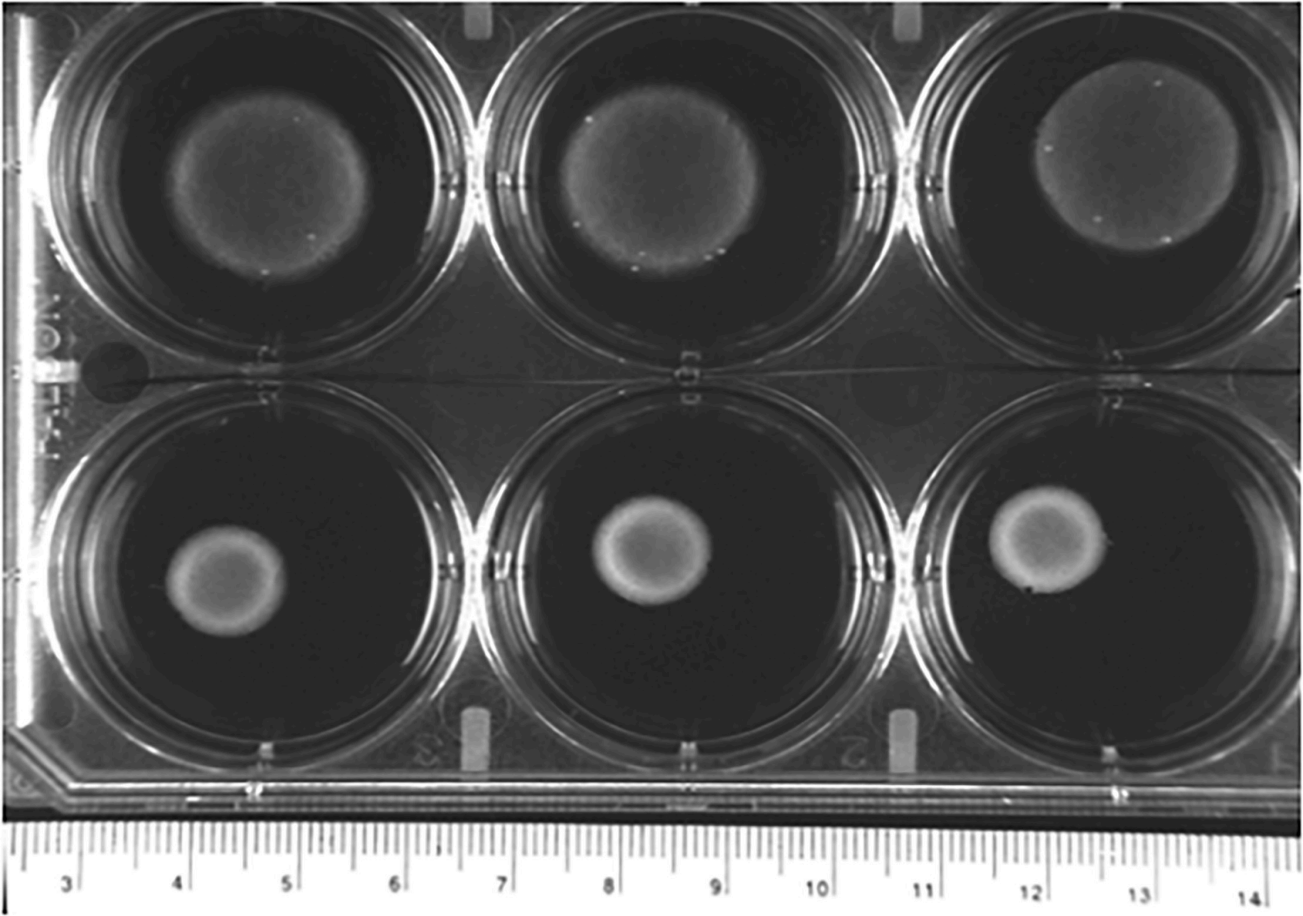
CPCG model system. CPCGs are seen floating in 6-well dish, with in-picture ruler for scale. The effects of HD (bottom) and LD (top) cell number on the ability to contract collagen is seen by the reduction in size of the gel.

Digital images of the gels were taken daily with a Bio-Rad Chemi-Doc system (Bio-Rad Laboratories, Hercules, CA) with an in-picture centimeter ruler for scale (Fig 1). The images were imported into image analysis software (SigmaScan Pro, SPSS Science, San Jose, CA) and pixels/cm calibrations were made. Gel outlines were manually traced, and gel areas were calculated in square centimeters. Cell morphology in CPCGs was assessed by fluorescent microscopy and stereomicroscopy.

### Histology

CPCGs were fixed overnight in Histochoice MB (Electron Microscopy Sciences, Hatfield, PA) at 4° C. Samples were paraffin embedded (TP10150 Tissue Processor, Leica, Bannockburn, IL), and sectioned at 4 μm. Sections were stained for histology with hematoxylin and eosin (H&E) (ThermoFisher, Waltham, MA) or for immunohistochemistry for alpha-SMA, using an anti-alpha-SMA antibody directly conjugated to fluorescein isothiocyanate (1:200 dilution, Jackson ImmunoResearch,West Grove, PA), and then counterstained with 4,6-diamidino-2-phenylindole (DAPI, Vector Laboratories, Burlingame, CA).

### Reverse transcriptase PCR

Total cellular RNA was extracted and isolated from intact HD-CPCGs by TRIzol (Invitrogen, Carlsbad, CA) followed by preparation of cDNA using the SuperScript First-Strand Synthesis System (Invitrogen, Carlsbad, CA). RNA quantification and purity was determined by spectrophotometry. PCR was performed using REDExtract N-Amp PCR Ready Mix (Sigma-Aldrich, St. Louis, MO) for 30 cycles in a DNA thermocycler (Eppendorf, Hauppauge, NY) using specific primers for integrin receptor components alpha1, alpha2, alpha5, beta1, CD44, and for hyaluronan-mediated motility (RHAMM), alpha-smooth muscle actin (alphaSMA); and growth factors: bFGF, PDGF, VEGF, and SDF-1alpha. The PCR products were analyzed by gel electrophoresis and densitometry was measured using Image J analysis software (http://rsbweb.nih.gov/ij), and standardized to beta-actin.

### Statistical analysis

Statistical comparisons were performed using repeated-measures ANOVA. Statistical significance was indicated by p < 0.05. In certain cases, error bars were omitted from the Figs when they were either encompassed by the data point symbol itself, in order to clarify the presentation of the data.

## Results

### MSC contract the gel more efficiently than DF

Our results show that MSC cultured at LD induced a significant contraction of the gel (Fig 1, upper panel). However, higher density cultures were able to contract the gel almost completely (Fig 1, lower panel).

**Fig 2:**
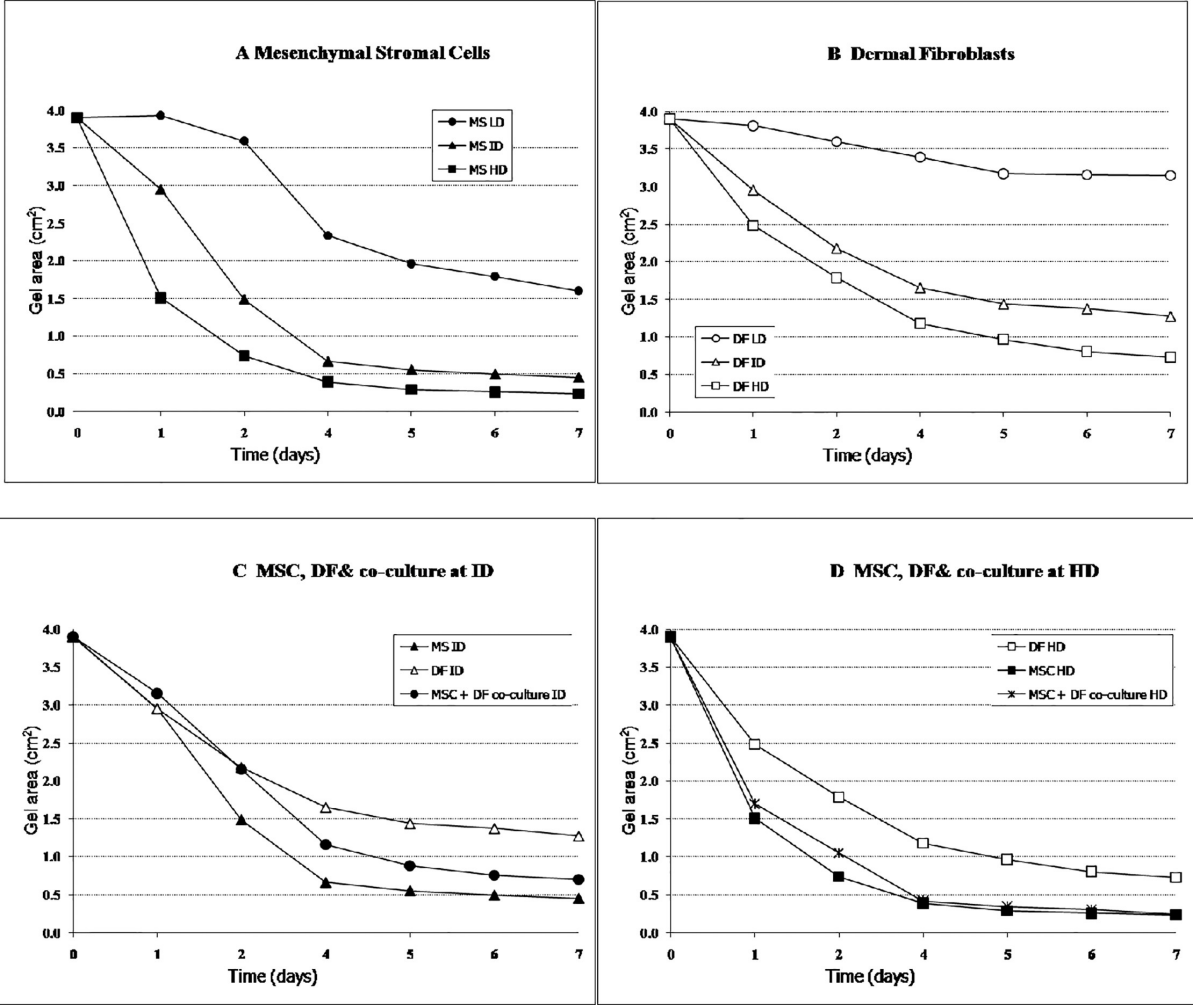
Contraction dynamics of CPCG. Contraction of MSC-populated collagen gels seeded at one of three cell densities, illustrating a sigmoid curve featuring initial “lag phase” of minimal macro-contraction, followed by “log phase” of rapid contraction. Increased cell density reduced lag phase (shifting curve to the left), increased rate of log phase contraction, and increased overall contraction at 7 days (2A). Contraction dynamics of DF-populated collagen gels seeded at one of three cell densities (2B). Contraction dynamics of co-cultured MSC/DF-populated collagen gels at 1.00 x 10^5^ cells/ml total cell concentration (2C) and at 2.00 x 10^5^ cells/ml total cell concentration (2D). Each graph represents single experiment in quadruplicate (error bars are within the bound of the symbols and thus not shown).

Fig 2A and 2B respectively, show a comparison between the effect of different densities of MSC and DF on gel contraction. Our results show that MSC at LD, ID, and HD induced greater contraction measured by the gel area from day 0 to day 7. CPCGs contraction curves were essentially sigmoidal, featuring an initial “lag phase”, the period of time before significant macro-contraction of the gel begins, followed by a “log phase” contraction, a period of rapid reduction in gel size. Increased cell density reduced the “lag phase”, shifting the contraction curve to the left, and increased both the rate of “log phase” contraction (as indicated by the slope of the contraction curve) and the overall contraction after 7 days.

### MSC interact with DF

**Fig 3:**
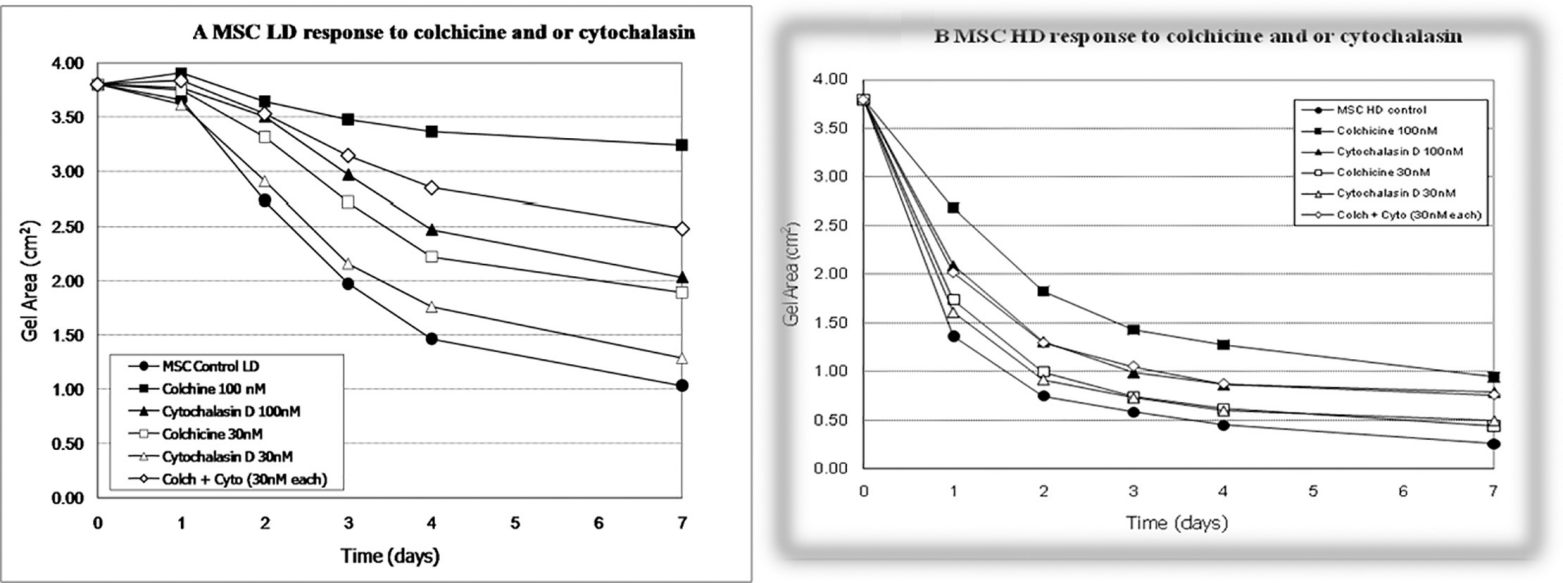
A) Contraction of LD-MSC gels (5.00 x 10^4^ cells/ml) in response to two concentrations of colchicine (30 nM, 100nM), two concentrations of cytochalasin D (30nM, 100 nM), or in combination (30nM of each). B. Contraction of HD-MSC gels (2.00 x10^5^ cells/ml) in response to two concentrations of colchicine (30nM, 100nM), or in combination (30nM of each). (Each graph represents single experiment in quadruplicate (error bars are within the bound of the symbols and thus not shown).

In order to determine if MSC have any sort of interaction with DF, we carried out co-cultures of MSc and DF in CPCGs and we looked at gel contraction. Our results show that gels containing both types of cells manifested more contraction than DF alone but less than MSC alone at an intermediate density (Fig 3A and 3B). However, when we increased the density of cells, MSC and MSC+DF induced same level of contraction (Fig 2).

### Inhibition of contraction

As shown in Fig 3, both colchicine and cytochalasin-D dose-dependently inhibited contraction of LD (Fig 3A) and HD (Fig 3B) MSC gels. The combination of low concentrations of colchicine and cytochalasin-D (30 nM each) resulted in additive inhibition of contraction. Higher concentrations of cytoskeletal disruptors completely blocked MSC-mediated contraction (10^−6^ M for LD gels, 10^−5^ M for HD gels; not shown). Specifically, in LD gels, colchicine and cytochalasin-D extended the lag phase, i.e. inhibited the onset of log phase contraction, and reduced both the log phase contraction rate and overall contraction (Fig 3A). By day 2, control LD gels had begun log phase and had contracted to 72% of original area; gels incubated in 100nM colchicine, 100nM cytochalasin-D, or 30/30nM colchicine/cytochalasin-D exhibited significantly reduced contraction (96, 92, and 93% of original area, respectively). Similarly, after 4 days, control LD gels had contracted to 39% of original area, while gels incubated in 100nM colchicine, 100nM cytochalasin-D, or 30/30nM colchicine/cytochalasin-D exhibited significantly reduced contraction (89, 65, and 75% of original area, respectively). In terms of HD gels, 100nM of colchicine or cytochalasin-D delayed the onset of log phase contraction and reduced the rate of contraction during the log phase. After 24 hours, control HD gels had contracted to 36% of original area, while gels incubated in 100nM colchicine, 100nM cytochalasin-D, or 30/30nM colchicine/cytochalasin-D exhibited significantly reduced contraction (71, 55, and 53% of original area, respectively). After 4 days, control HD gels had contracted to 12% of original area, while gels incubated in 100nM colchicine, 100nM cytochalasin-D, or 30/30nM colchicine/cytochalasin-D exhibited significantly reduced contraction (34, 23, and 23% of original area, respectively).

### Contraction in response to conditioned media and/ Growth factors

**Fig 4:**
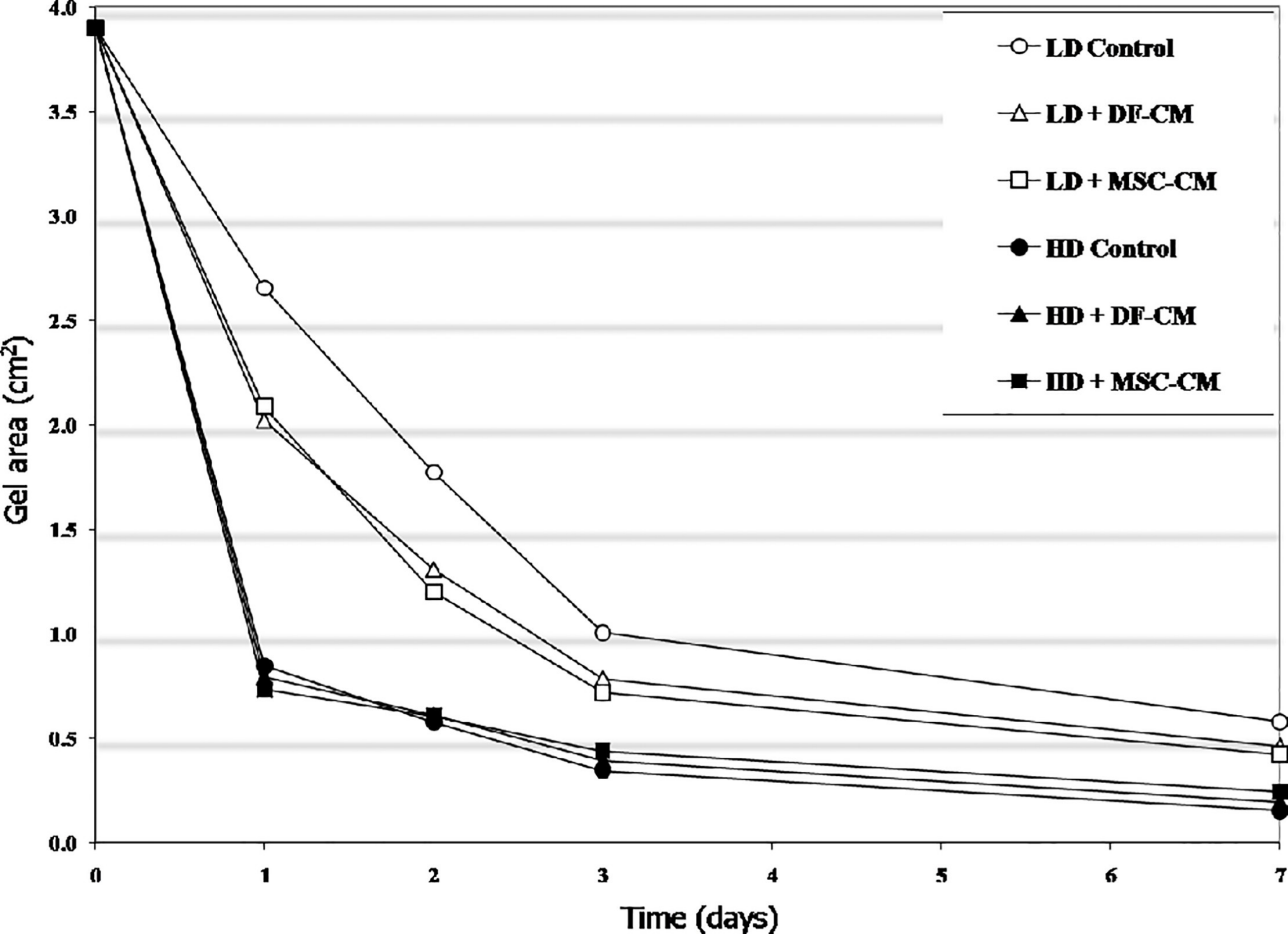
Contraction dynamics of LD and HD MSC-populated collagen gels incubated with normal MGM (control), MSC-, or DF-conditioned media. Graph represents single experiment in quadruplicate (error bars are within the bound of the symbols and thus not shown).

**Fig 5:**
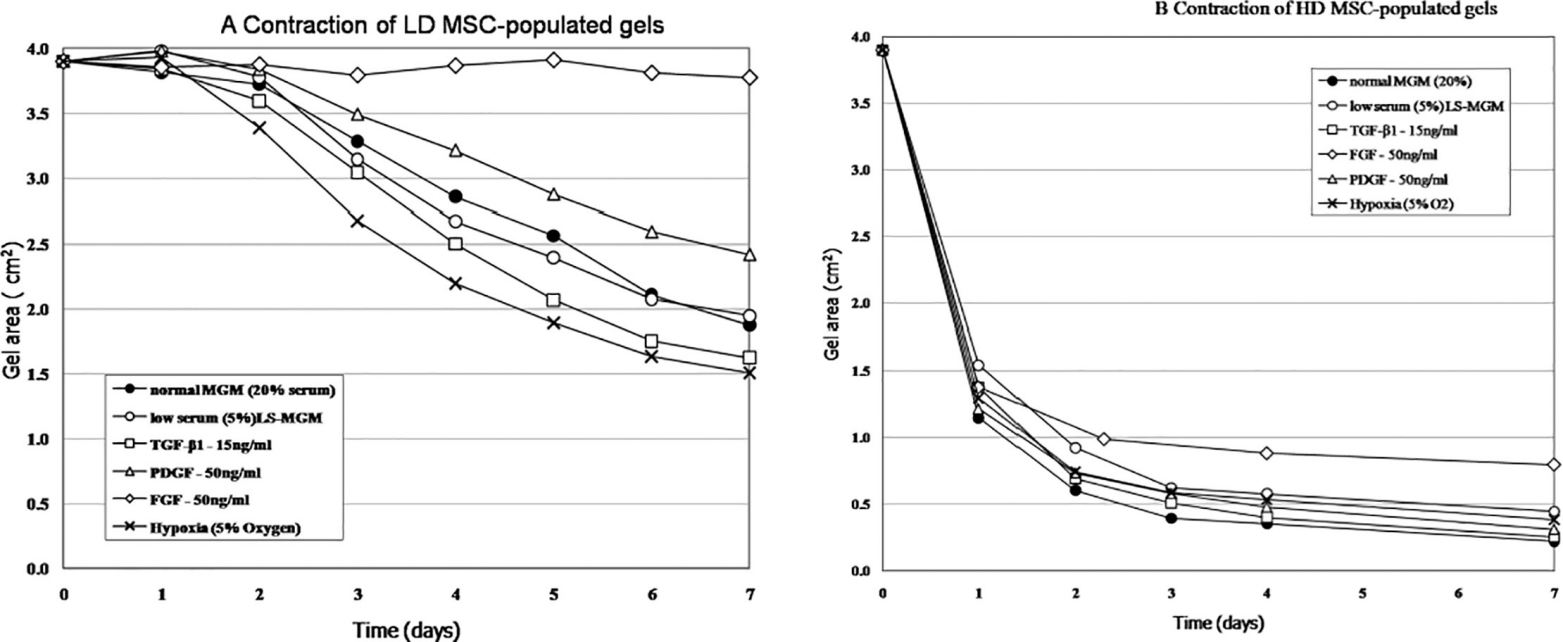
Contraction dynamics of LD (5A.) and HD (5B.) MSC-populated collagen gels incubated with low-serum media (LS-MGM), normal MGM, listed growth factors (FGF, TGF-B1, PDGF-BB), or under hypoxic conditions (vs. normoxic; 5% O_2_). Each graph represents single experiment in quadruplicate (error bars are within the bound of the symbols and thus not shown).

To determine the effect of soluble mediators that may be secreted into the wound environment, LD and HD MSC gels were incubated in either MSC or DF conditioned media. As shown in Fig 4 both MSC- and DF-conditioned media significantly enhanced the contraction of LD gels. At day 2, control gels averaged 39% of original area, compared to 28% and 25% of original area for gels incubated in MSC- or DF-conditioned medium, respectively. No significant differences on contraction were seen between MSC- and DF-conditioned medium. The effect of conditioned media on HD-MSC gels was less pronounced compared to LD-MSC gels. As shown in Fig 5, in LD MSC gels, TGFb1 enhanced contraction, while PDGF decreased and FGF completely inhibited contraction (Fig 5A). In HD MSC gels, TGFb1 and PDGF slightly enhanced, and FGF slightly inhibited contraction (Fig 5B).

Many chronic wounds characterized by tissue loss have an element of hypoxia associated with them. To determine the effects of hypoxia, gels were incubated at 5% O_2._ Hypoxia increased the contraction of both LD- and HD-populated gels (Fig 5A & 5B).

**Fig 6:**
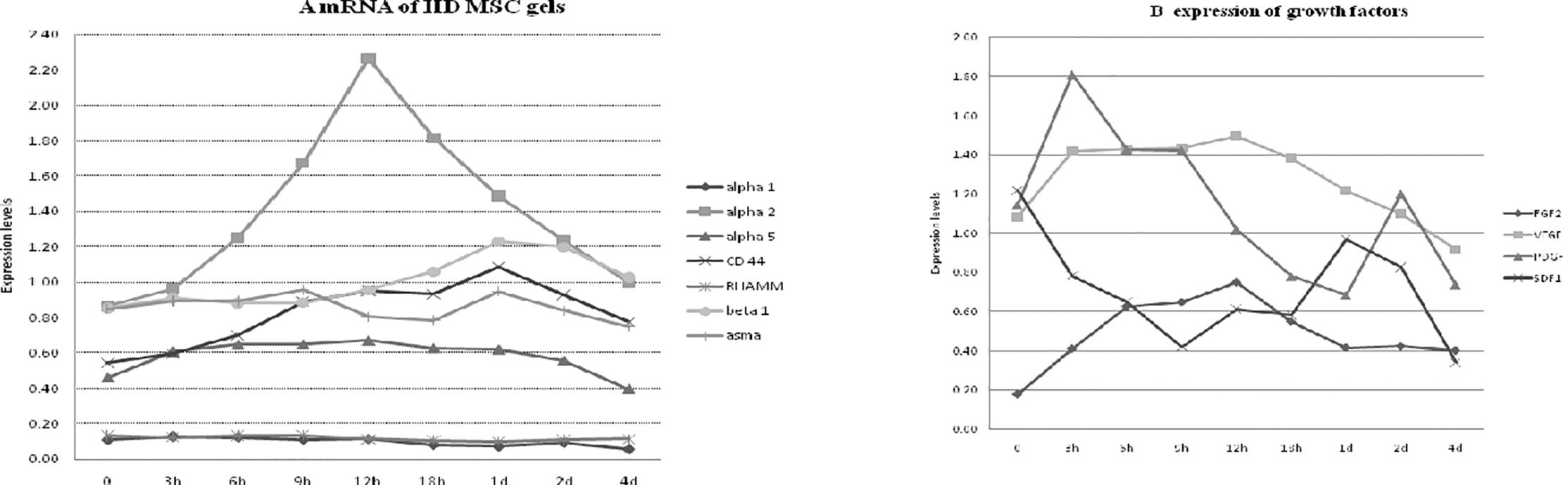
RT-PCR analysis of mRNA levels from gels seeded with MSC at high density over time. Panel A demonstrates expression of genes associated with ECM integrin receptor components, ∂-SMA, and RHAMM commonly expressed in fibroblasts or myofibroblasts. Panel B demonstrates growth factor gene expression commonly detected during wound healing.

Fig 6 outlines the results for the RT-PCR from intact HD MSC-CPCGs. As depicted in Fig 6A, mRNA levels of integrin alpha2 rapidly increases during the first 12 hours, remains elevated through 24 hours, and slowly decreases to, and remains at base level (day 0) from day 1 to day 4. Similarly, integrin beta1 and CD44 message increases during the first 2 days and decreases to base level by day 4. The alpha-SMA and integrin alpha5 mRNA levels remain relatively unchanged from day 0 to 4. Low, but constitutively expressed, levels of integrin apha1 and RHAMM were seen. As depicted in Fig 6B, MSC in collagen gels expressed FGF2, VEGF, PDGF, and SDF-1alpha. PDGF expression was bimodal, and featured an early peak at 3 hours, decreasing, but elevated expression from 3 hours to 12 hours, and a later peak at 2 days. FGF2 and VEGF mRNA expression increased during the first 12 hours, and returned to baseline levels by day 4. Following an initial decrease during the first 24 hours, SDF-1alpha mRNA expression showed a brief peak at 2 days.

### Morphology and alpha smooth muscle actin as a mechanism of contraction

**Fig 7:**
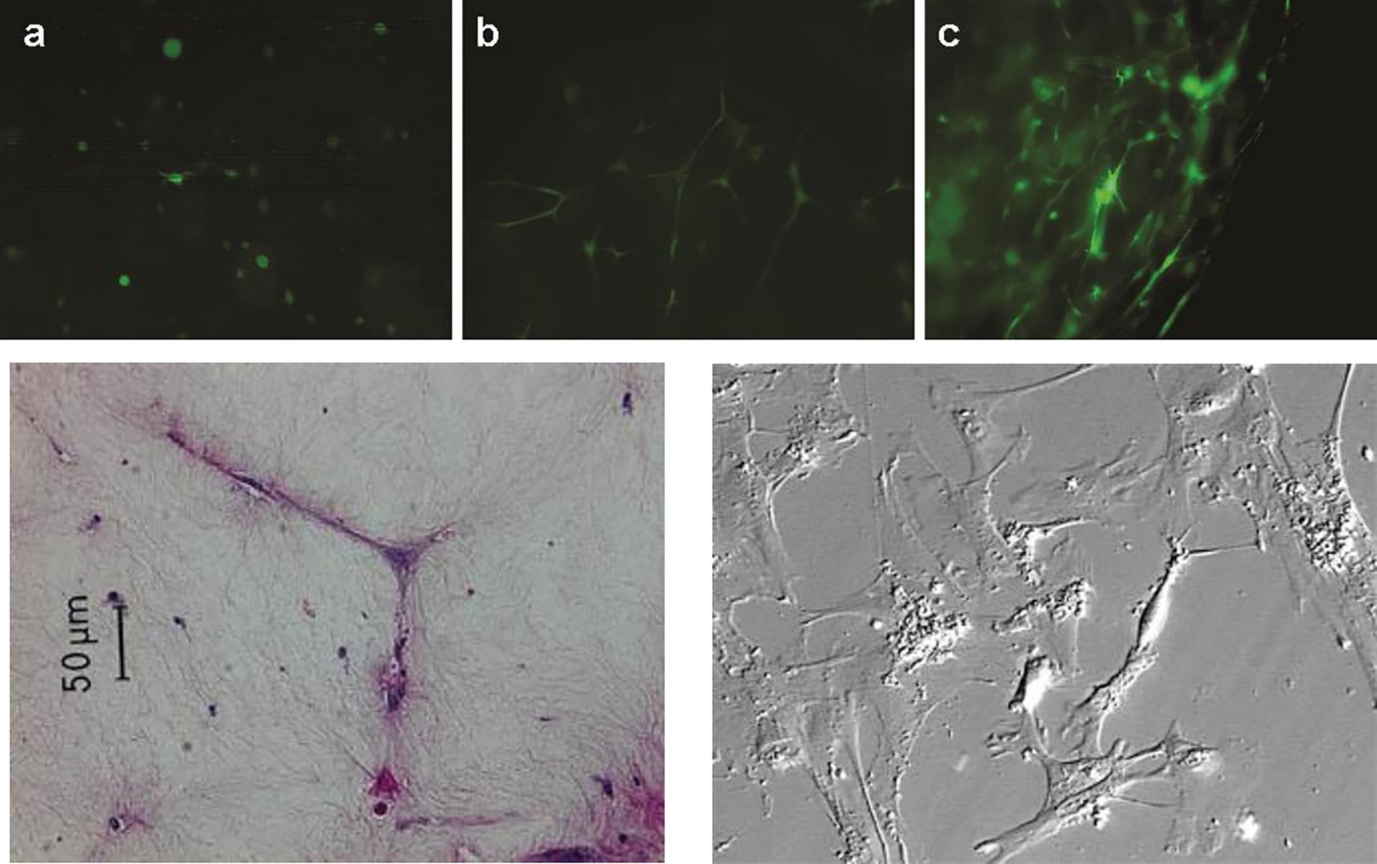
Fluorescent microscopic images of GFP-labeled MSC within gels after 3 days. (7A) MSC demonstrate a small round phenotype upon initial seeding with collagen gels. LD gel at 100X after 3 days, (7B). HD gel at 50X after 3 days. (2C). At 6 days, multiple cellular processes extend from individual cells to coalesce forming an extensive lattice network.

After trypsinization and incorporation into three-dimensional polymerized collagen gels, MSC rapidly spread, branched, and elongated into the collagen matrix (Fig 7A–7C). Initially cells demonstrated a small round morphology (Fig 7A) but by day 1 individual MSC begin to put forth cellular processes which are very evident after 3 days (Fig 7B). By day 6, all MSCs contributed to a dense lattice network, and each cell featured several markedly long, branched processes (Fig 7C).

## Discussion

The relative physiologic contributions of resident, nonresident or transplanted cells to accomplish wound repair have been a long-standing areas of both interest and controversy. Autologous bone-marrow cells, circulating stromal cells, and fibrocytes, to name a few, have all been implicated as contributors to skin repair, but the extent to which these cells directly participate in the biomechanical aspects of wound closure is unclear [22–29]. *In vitro* models, such as CPCG contraction, can provide unique, inexpensive, and reproducible methods to investigate cell behavior, biomechanics, and cell-matrix interactions in a three-dimensional environment similar to that *in vivo*. They allow for the isolation of individual components (e.g. cells or soluble factors) and mechanisms (e.g. cytoskeleton-mediated contractility or collagen binding and translocation) in real-time without the myriad of factors that often confound animal and clinical trials. These results demonstrated that, similar to fibroblasts, MSC contract collagen gels in a dose-dependent manner and that contraction dynamics are affected by soluble mediators and hypoxia, and in response to both serum and DF-conditioned media. MSCs contracted gels more efficiently than age-matched, fetal dermal fibroblast controls. Based on these results it seems reasonable to conclude that in addition to ‘indirect’ effects (autocrine and paracrine effects of synthesized soluble factors and matrix proteins), transplanted MSC are likely to interact with the wound ECM, primarily composed of fibrin and collagen types I and III, and to biomechanically contribute to wound contraction and closure [17–19,30]. Analysis of the dynamics of MSC-mediated contraction, as well as the influence of cell concentration, soluble mediators, and cytoskeletal elements allowed elucidation of these direct mechanisms.

During the initial lag phase of gel contraction, cells begin to spread and elongate, binding to collagen and extending filopodia and/or lamellipodia into the matrix. The data infers a reduced lag phase in MSC-mediated contraction, compared to DF-mediated; but more definitive analysis, that is measuring HD gel contraction areas every several hours, would confirm this inference. Nevertheless, these results suggest an enhanced inherent capacity, speed, and/or propensity of MSC compared to DF, to recapitulate and generate new cytoskeletal structures and integrins, and begin to bind and remodel collagen. Since fibroblasts in floating gels have been shown to become quiescent [31, 32], differences in proliferation likely does not explain these differences. During the log phase of the contraction curve, cells rapidly contract gels. The dynamics suggest that MSC have either inherently different, or differentially distinct, mechanisms of CPCG contraction compared to DF, or that MSC simply have enhanced efficiency and speed of collagen binding and translocation.

The etiology of chronic and diabetic wound healing impairments is multifactorial. Alterations in cytokine production, matrix synthesis and modification (e.g. glycosylation), cellular insensitivity or hypersensitivity to inflammatory mediators, dysfunctions in angiogenesis, and impaired cell proliferation, contractility, and motility have all been implicated as detrimental influences on normal healing in diabetic wounds [33–36]. Therapeutic approaches that focus on single aspects of impaired healing, e.g. topical PDGF, are typically insufficient and have demonstrated only limited successes. Cell therapy provides broad, multifarious, often synergistic benefits to problem wounds. MSC therapy, in particular, has shown numerous advantages compared to other cell lineages [37]. Autologous MSC are easier to obtain and rapidly expand to generate sufficient cell numbers for clinical use compared to dermal fibroblasts. This is especially relevant in diabetic patients, since it would be especially disadvantageous in this population to create a new dermal injury that may result in the same abnormal or delayed healing response.

In addition to the improved healing by MSC cytotherapy alone, our data suggest that the potential for clinical control and modulation of healing by the addition or variation of several factors such as alteration of oxygen tension or systemic or local application of cytokines or chemokines can further modulate MSC response based on cell number. First, the number of transplanted cells as an experimental variable has not been sufficiently explored, but normal wound healing features dramatic and dynamic temporal and spatial changes in the cellular profile and density of a number of cells, as well as their presumed levels of contractility, motility, and production of growth factors and matrix [38]. This research supports the hypothesis that LD gels contract through different primary mechanisms than those at HD [20, 34]. For example, a given dose of cytochalasin or colchicine (even in a ‘molecule-per-cell’ sense) affected LD CPCG contraction more dramatically than HD CPCG contraction. Similarly, the effects of CM, specific growth factors, and hypoxia were more pronounced in LD gels. Future cytotherapy research would be amiss to overlook this variable. Second, local application of normal or genetically-modified MSC in combination with exogenous protein may represent a powerful strategy for the promotion of wound closure. The specific factor(s) that contributed to CM-mediated increased contraction still need to be identified. The effect of conditioned medium was apparent even though the CM was ‘old,’ and had presumably lost some of its potency, and the high percentage of serum in Mesencult (20%) could have partially masked some of its effects. Regardless, soluble factors secreted by DF influenced MSC-mediated collagen contraction and migration. Specifically, TGF-beta1 slightly increased MSC-mediated contraction, an effect that has also been seen in fibroblasts [39–42], but interestingly, PDGF and FGF inhibited contraction. Since the data suggests that SDF-1alpha is down regulated during initial cell elongation into the matrix and late log phase contraction, a similar initial scenario could be envisioned *in vivo*, in that exogenous SDF-1alpha may be required to enhance the recruitment of other healthy autologous cells. Third, cytoskeletal elements have long been therapeutic targets for enhancing wound healing, modulating contraction, and inhibiting scar contracture, but the results have been mixed [43–46]. The cytoskeleton is vital to cell motility and contractility, and as previously described with fibroblasts [20, 43], MSC-mediated CPCG contraction involves differential and complementary contributions of both microtubules and microfilaments. Judicious, transient use of specific, targeted cytoskeletal disruptors may further enhance the benefit of MSC therapy in a variety of wound healing applications. Finally, it is known that wounds are hypoxic, albeit less so than the level of hypoxia we evaluated in this study. Considering our data, it is reasonable to believe that modulation of oxygen tension in wounds may influence the contractility of resident or transplanted cells.

What is less clear is the clinical implication of CPCG contraction since collagen gels do not recapitulate the complex granulating wound environment characterized by a more extensive array of extracellular matrix proteins containing multiple cell types. Specifically, if a given cell type demonstrates increased contraction in the CPCG model, does that correlate to improved *in vivo* contraction or enhanced wound closure? Kuhn et al. found no correlation between contraction of pressure ulcer-derived fibroblast-populated collagen gels and healing [47]. Viennet et al. showed that fibroblasts from chronic venous leg ulcers, presumably deficient in some way, contracted gels more rapidly than those from age-matched controls, notably those fibroblasts obtained from the center of the ulcers [48]. However, normal wound fibroblasts, a population with presumably more activated fibroblasts and myofibroblasts, were shown to contract collagen gels to a greater extent than normal dermal fibroblasts [49]. Badillo et al. showed that transplanted GFP-expressing MSC cells co-expressed alpha-SMA, suggesting that after transplantation, some or all MSC may differentiate *in situ* to express a myofibroblast-like phenotype [15]. Here, MSC in CPCG expressed alpha-SMA, and in HD-CPCG, alpha-SMA mRNA was constitutively expressed. However, mRNA levels remained relatively constant throughout log phase contraction. Regardless, these results infer that MSC can exert biomechanical influences in the wound environment, and this alone may offer significant clinical advantages, particularly when wound closure is of primary importance.

## Supporting information

S1 File Fig 2 data. - supporting data for Fig 2. (XLS)

S2 File Fig 3 data + extra. - supporting data for Fig 3. (XLS)

S3 File Fig 4 data. - supporting data for Fig 4. (XLS)

S4 File Fig 5 data. - supporting data for Fig 5. (XLS)

S5 File Fig 6 data.- supporting data for Fig 6. (XLS)

S6 File Fig 8 data. - supporting data for Fig 7, note the figure numbing was changed in the final edits. (XLS)
